# Long-term survival and late complications of intensity-modulated radiotherapy for recurrent nasopharyngeal carcinoma

**DOI:** 10.1186/s12885-018-5055-5

**Published:** 2018-11-20

**Authors:** Fangfang Kong, Junjun Zhou, Chengrun Du, Xiayun He, Lin Kong, Chaosu Hu, Hongmei Ying

**Affiliations:** 1 Department of Radiation Oncology, Fudan University Shanghai Cancer Center, Shanghai, People’s Republic of China; 2 Department of Oncology, Shanghai Medical College, Fudan University, Shanghai, People’s Republic of China; 3 Department of Radiation Oncology, Renji Hospital, School of Medicine, Shanghai Jiao Tong University, Shanghai, People’s Republic of China; 4 Department of Radiation Oncology, Shanghai Proton and Heavy Ion Center, Fudan University Shanghai Cancer Center, Shanghai, People’s Republic of China

**Keywords:** Intensity-modulated radiotherapy, IMRT, Recurrent nasopharyngeal carcinoma, NPC, Re-irradiation, Survival, Late complication

## Abstract

**Background:**

To evaluate the effectiveness and toxicities of intensity-modulated radiotherapy (IMRT) for locally recurrent nasopharyngeal carcinoma (NPC).

**Methods:**

One hundred and eighty-four previously irradiated NPC patients with recurrent disease and re-irradiated by IMRT between February 2005 to May 2013 had been reviewed. The disease was re-staged I in 33, II in 27, III in 70 and IV in 54 patients. Seventy-five percent of the patients received cisplatin-based chemotherapy.

**Results:**

The median survival time was 33 months. The 3-year actuarial rates of local recurrence–free survival (LRFS), distant metastases–free survival (DMFS), and overall survival (OS) rates were 85.1, 91.1, and 46.0%, respectively. About 53% of the patients experienced Grade 3–4 late toxicities. Forty-four patients died of massive hemorrhage of the nasopharynx caused by radiation induced mucosal necrosis. Multivariate analysis indicated that chemotherapy and time interval between initial radiotherapy and re-irradiation were independent predictors for DMFS.

**Conclusion:**

IMRT is an effective method for patients with locally recurrent NPC. Massive hemorrhage of the nasopharynx is the major sever late complication and also the leading cause of death. Early recurrence is negative factor for DMFS. Combination of chemotherapy can improve DMFS, but not for OS. Optimal salvage treatment strategies focusing on improvement of survival and minimization of late toxicities are warranted.

## Background

Recurrent nasopharyngeal carcinoma (NPC) occurred in 20–40% for patients treated with traditional radiotherapy [1–3]. With the development of modern radiation technique, the local control has been definitely improved. However, recurrence inevitably happens in some patients, especially those with local-regionally advanced disease [4]. Salvage treatment for locally recurrent NPC remains a challenge for clinical oncologists. Various strategies, including surgery, brachytherapy, and stereotactic radiosurgery have been used in attempt to cure locally recurrent NPC in the past several decades [5–7]. However, their utility is usually limited by the extent of disease at recurrence. It is reported that 70–80% of the recurrent NPC were locally advanced [8–11]. For patients with infiltrative or extensive disease, definitive reirradiation is an important way of treatment. Since curative re-irradiation with conventional techniques is associated with a considerable risk of severe complications, including temporal lobe necrosis, trismus, mucosal ulcer and even fatal hemorrhage, the treatment planning is often difficult and poses a special challenge to radiation oncologists [12].

Intensity-modulated radiotherapy (IMRT) is an ideal radiation modality for NPC, due to its favorable balance between target coverage and the sparing of adjacent organs [13]. Published reports have shown that the use of IMRT for retreatment of locally recurrent NPC is clinically feasible and could produce acceptable disease control [8, 9, 12, 14, 15]. However, reports of long-term results with large samples were relatively rare. Herein, we reported our institutional experience of IMRT for recurrent NPC in 184 patients.

## Methods

### Patients

From February 2005 to May 2013, 184 patients with recurrent NPC treated by curative re-irradiation with IMRT in Fudan University Shanghai Cancer Center were enrolled in this study. All patients received complete assessment of history and physical examination, contrasted magnetic resonance imaging (MRI) of the nasopharynx and neck, chest computed tomography (CT) or radiography, abdominal ultrasound, endoscopy, complete blood test and emission-computed tomography (ECT) if necessary. All patients were re-staged according to the 2010 American Joint Committee on Cancer/Union for International Cancer Control staging system (AJCC/UICC 2010). This study was approved by the Institutional Review Boards of Fudan University Shanghai Cancer Center, China. Written informed consent was obtained from the patients before treatment. Due to the retrospective design of the study, the local ethic committee confirmed that informed consent was not necessary from participants.

### Treatment of recurrence

#### Intensity-modulated radiotherapy

Patients received CT simulation at 3-5 mm thickness with custom head mask in the supine position. Contrasted MRI and CT image fusion was performed for target delineation. The gross tumor volume (GTV) included all recurrent tumors seen on diagnostic CT/MRI, endoscope and physical examinations. The clinical target volume (CTV) was defined as the GTV plus 5 to 10 mm margin to encompass any microscopic extension. The planning target volume (PTV) would encompass the CTV/GTV with a 3-5 mm margin in all directions.

Six-MV photons and simultaneous integrated boost (SIB) technique were used to treatment planning. The median dose was 66.7Gy (range, 42-77Gy). The fractional dose was 1.8–2.3Gy/day (5 days per week). Thirteen patients received brachytherapy boost.

#### Chemotherapy

Cisplatin-based neoadjuvant, concurrent, and/or adjuvant chemotherapy were administrated in patients with locally advanced disease or with a relatively short interval between the end of primary RT and tumor recurrence. Seventy-seven (41.8%) patients received neoadjuvant chemotherapy, 46 (25%) patients received IMRT alone. Nineteen patients received targeted agents (cetuximab or nimotuzumab). Details were shown in Table 1.

**Table 1:** Treatment schedule for patients with locally recurrent NPC

Treatment schedule	No. (%)
Chemotherapy	
Neo + RT	77 (41.8)
Neo + CCRT	23 (12.5)
Neo + RT + Adj	17 (9.2)
CCRT	10 (5.4)
CCRT+Adj	4 (2.2)
Neo + CCRT+Adj	2 (1.1)
RT + Adj	5 (2.7)
IMRT alone	46 (25)
Cetuximab	9 (4.8)
Nimotuzumab	10 (5.4)
IMRT	
IMRT dose (Gy)	
Median (range)	66.7 (42–77)
Fractional dose(Gy)	
1.8	1 (0.5)
2.0	125 (67.9)
2.1	44 (23.9)
2.2	6 (3.3)
2.3	7 (3.8)
IMRT treatment duration (days)	
Median (range)	46 (29–64)
Brachytherapy	13 (7.1)

Abbreviations: *NPC*, nasopharyngeal carcinoma; *Neo*, neoadjuvant chemotherapy; *RT*, radiotherapy; *CCRT*, concurrent chemoradiotherapy; *Adj*, adjuvant chemotherapy; *IMRT*, intensity-modulated radiotherapy

### Patient evaluation and follow up

Tumor response and toxicities were assessed weekly during IMRT, every 3–6 months after treatment in the first 5 years, and yearly thereafter. Imaging examinations (MRI, CT and ultrasound) were performed 3 months after IMRT and then every 6–12 months. The final date of follow-up was October 12, 2016.

Treatment-related toxicities were graded according to National Cancer Institute Common Toxicity Criteria for Adverse Events (NCI-CTCAE) version 4.02.

### Statistical analysis

The rates of local recurrence–free survival (LRFS), distant metastases–free survival (DMFS) and overall survival (OS) were estimated with the Kaplan-Meier method. The durations were calculated from the date of diagnosis of local recurrence to the date of each event occurred or the last follow-up. Log-rank tests and Cox proportional hazards model were used for univariate and multivariate analysis respectively. The level of significance was set at a *P* value less than 0.05. Statistical analyses were carried out with the Statistical Package for Social Sciences software (SPSS v16.0; IBM Corporation, Armonk, New York, USA).

## Results

### Patient characteristics

Patient characteristics were detailed in Table 2. The median age at the time of re-irradiation was 49 years old. About 65% of the patients were rT3–4 disease. Thirty-one patients had synchronous nodal recurrence. Patients were previously treated with two-dimensional conventional radiotherapy (RT) or IMRT to a median dose of 70Gy (range, 36–78.35Gy). Six patients received brachytherapy for local residual disease. Cisplatin-based chemotherapy was given for patients with local and/or regional advanced disease. Forty-four patients received RT alone. The median time between initial radiotherapy (RT) and recurrence was 35 months (range 6–388 months).

**Table 2:** Characteristics of patients with locally recurrent NPC treated with IMRT

Characteristic	No. (%)
Gender	
Male	133(72.3)
Female	51(27.7)
Age at the time of re-irradiation (years)	
Median (range)	49(23–86)
Primary RT technique	
2D-CRT	103 (56)
IMRT	43 (23.4)
Unknown	38 (20.7)
Primary RT dose (Gy)	
Median (range)	70 (60–78.35)
Chemotherapy in the first treatment	
Yes	103 (56)
No	44 (23.9)
Unknown	37 (20.1)
rT classification	
rT1–2	64 (34.8)
rT3–4	120 (65.2)
Presence of synchronous nodal recurrence	
No	153 (83.2)
Yes	31 (16.8)
Time interval between initial RT and recurrence (months)	
Median (range)	35 (6–388)
TTR (years)	
≤ 2	59 (32.1)
> 2	125 (67.9)

Abbreviations: *NPC*, nasopharyngeal carcinoma; *IMRT*, intensity-modulated radiotherapy; *RT*, radiotherapy; *2D-CRT*, two-dimensional conventional radiotherapy; *TTR*, time to recurrence

Local recurrence was diagnosed by biopsy and/or CT/MRI/PET-CT evidence of progressive skull base erosion and clinical symptoms. One hundred and thirty-six (73.9%) patients were diagnosed by biopsy. Forty-eight (26.1%) patients had deep-seated recurrences in the skull base and/or intracranial, and were diagnosed by CT/MRI/PET-CT and clinical symptoms.

All patients completed their planned radiation except for 5 patients who prematurely terminated their treatment because of acute side effects and/or personal reasons after receiving doses between 42-64Gy.

### Survival

**Fig. 1:**
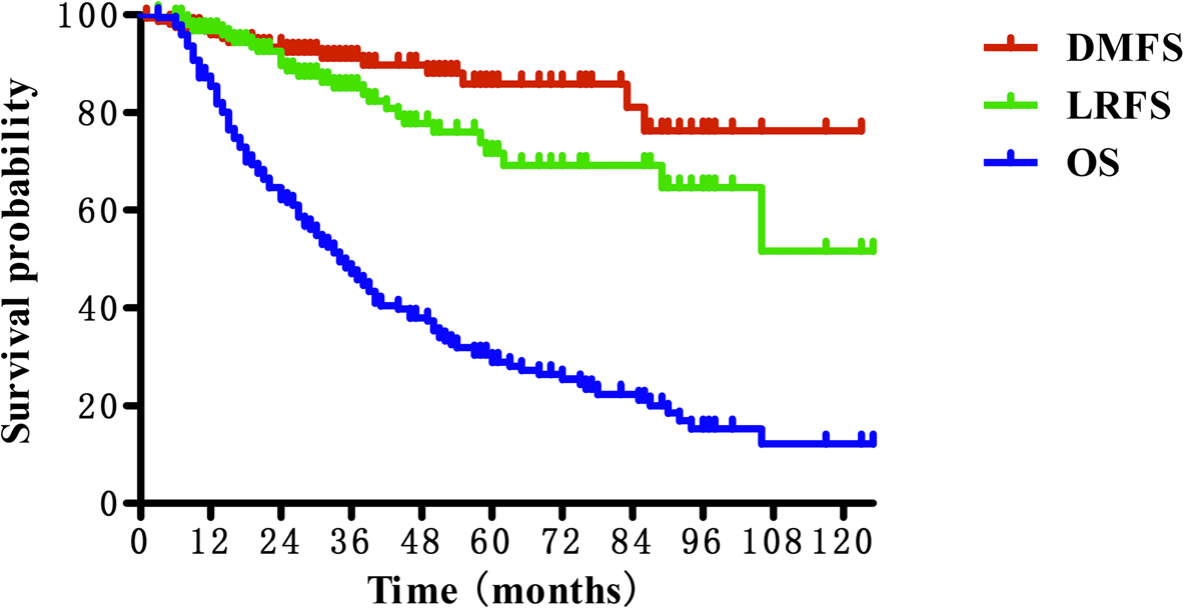
Kaplan-Meier curves showing local recurrence-free survival (LRFS), distant metastases-free survival (DMFS) and overall survival (OS) for patients with recurrent nasopharyngeal carcinoma

The median follow-up time was 32 months (range 3 to 125) for the entire group, and 68 months (range 10 to 125) for the survivors. Eight patients were lost to follow-up. Recurrence was observed in 43 (23.4%) patients. Among them, 27 patients failed in local, 14 patients failed in regional, and 2 patients failed both in local and regional. Sixteen (8.7%) patients suffered distant metastasis. Common sites for distant metastasis were lung (9 patients), bone (5 patients) and liver (4 patients). The 3-year LRFS and DMFS rates were 85.1 and 91.1%, respectively. And the 5-year LRFS and DMFS rates were 71.7 and 85.9%, respectively (Fig. 1).

At the time of analysis, a total of 134 patients (72.8%) died. The median survival time was 33 months. The 3 and 5-year OS rates was 46.0 and 28.8% (Fig. 1). The cause of death was disease progression in 39 patients, radiation-induced injuries in 54 patients (including profuse epistaxis in 44 patients, feeding difficulty in 3 patients and other injuries in 7 patients) and unknown causes in 36 patients. And the remaining 5 patients died of infection or cardio-cerebrovascular disease.

### Toxicities

Late severe adverse effects (SAEs) (≥grade 3 toxicities) were recorded after IMRT (Table 3). Common late SAEs included mucosal necrosis, headache, cranial nerve palsy, trismus. Forty-four (24.9%) patients with mucosal necrosis died of profuse epistaxis since the necrosis involved the internal carotid artery. The median latency for mucosal necrosis was 6.0 months (range 0.5–65.5 months) after re-irradiation.

**Table 3:** Late severe toxicities for patients with locally recurrent NPC

Toxicities	Grade 3–4 No. (%)	Grade 5 No. (%)
Mucosal necrosis	12(6.8)	44(24.9)
Headache	16(12.8)	0
Cranial nerve palsy	21(11.9)	0
Temporal lobe necrosis	0	0
Trismus	35(19.9)	0
Hearing deficit	10(5.6)	0
Neck fibrosis	1(0.6)	0

### Prognostic factors

Univariate and multivariate analysis of potential factors including gender, age, rT stage, fractional dose, chemotherapy, time to recurrence (TTR), IMRT dose, and cumulative dose, brachytherapy for LRFS, DMFS and OS was performed. The results showed that chemotherapy (hazard ratio (HR), 0.210; 95% confidence interval (CI), 0.065–0.677; *P* < 0.01) and TTR (HR, 0.291; 95% CI, 0.089–0.949; *P* = 0.041) were independent predictors for DMFS. No significant prognostic factors were found for LRFS and OS. The details are shown in Tables 4 and 5.

**Table 4:** Univariate analysis of potential prognostic factors

Items	3-yr LRFS		3-yr DMFS		3-yr OS	
	%	p	%	p	%	p
Gender						
Male	90.1	0.356	89.3	0.387	41.8	0.980
Female	83.1		91.8		47.6	
Age (years)						
< 60	84.9	0.884	89.0	0.070	51.0	0.026
≥ 60	87.2		100		32.4	
rT stage						
rT1–2	96.6	0.070	94.7	0.362	51.8	0.241
rT3–4	77.9		88.8		42.9	
FD (Gy)						
≤ 2	81.6	0.298	90.3	0.820	45.5	0.645
> 2	94.0		93.2		47.4	
Use of Chem						
No	88.8	0.433	83.2	0.328	49.0	0.196
Yes	83.8		93.9		45.1	
TTR (years)						
≤ 2	91.7	0.248	83.9	0.035	43.6	0.977
> 2	82.1		94.8		47.0	
IMRT dose (Gy)						
< 70	85.6	0.790	93.6	0.908	42.8	0.652
≥ 70	84.7		86.1		54.0	
Cumulative dose(Gy)						
< 140	85.5	0.508	88.3	0.118	49.2	0.095
≥ 140	88.0		96.2		41.0	
Brachytherapy						
No	79.1	0.027	90.6	0.887	44.6	0.495
Yes	100		94.4		57.9	

Abbreviations: *LRFS*, local recurrence–free survival; *DMFS*, distant metastases–free survival; *OS*, overall survival; *FD*, Fractional dose; *Chem*, chemotherapy; *TTR*, time to recurrence

**Table 5:** Impact of prognostic factors on treatment results by multivariate analysis (*p* value)

Factors	LRFS	DMFS	OS
Age (years)			
< 60 vs. ≥60	–	0.970	0.106
rT stage			
rT1–2 vs. rT3–4	0.918	0.463	0.315
FD (Gy)			
≤ 2 vs. > 2	0.137	–	0.686
Use of Chem			
No vs. Yes	0.610	0.009	0.342
TTR (years)			
≤ 2 vs. > 2	0.964	0.041	–
IMRT dose (Gy)			
< 70 vs. ≥70	0.575	–	–
Cumulative dose (Gy)			
< 140 vs. ≥140	–	0.117	0.112
Brachytherapy			
No vs. Yes	0.051	–	–

Abbreviations: *LRFS*, local recurrence–free survival; *DMFS*, distant metastases–free survival; *OS*, overall survival; *FD*, Fractional dose; *Chem*, chemotherapy; *TTR*, time to recurrence

## Discussion

There is still no consensus on the treatment of locally recurrent NPC. Surgical resection can remove radio-resistant tumor and avoid re-irradiation related injuries for patients with early recurrent disease (rT1–2). It is reported that the 5-year local control (LC) and OS rates for nasopharyngectomy were 43–74% and 47–62%, respectively [7, 16–19]. However, most of the recurrent tumor was too advanced (rT3–4) at diagnose to be resected [6, 20–22]. For these patients, re-irradiation could be the only potential effective treatment. Re-irradiation with traditional radiation techniques is often associated with severe late complications and resulting in decreased quality of life.

IMRT has been widely used in primary NPC and achieved exciting LC, survival and tolerable toxicities [23–28]. As to recurrent NPC, IMRT may potentially help to improve LC and reduce toxicities. Hsiung et al. [29] compared 3D-CRT and IMRT for the boost or salvage treatment of NPC, and found that IMRT plans can achieve lower normal tissue does and more homogeneous target doses. Several studies have shown satisfactory long-term results of IMRT for recurrent NPC, with a 3-year local control and overall survival rates of 70–89% and 46–58%, respectively [8–10]. In the present study, we observed a 3-year LRFS and OS of 85.1 and 46.0%, respectively, which were comparable with other reports. Our results further confirmed that IMRT is an effective choice of treatment for patients with recurrent NPC.

As the local control and survival rates improved, quality of life (QOL) is increasingly emphasized. Radiation-related severe late complications are negative factors affecting QOL. In the study by Hua et al. [8], grade 3–4 late toxicities occurred in 34.4% of the patients after re-irradiation with IMRT for recurrent NPC. About 30% of the patients died of excessive nasal bleeding caused by mucosal necrosis. And they also found that the incidence of severe late complications were higher in patients with advanced disease than with early-stage disease. Chan and colleagues [4] recently reported their treatment results of IMRT for recurrent T3–4 NPC in 38 patients. The 3-year local control rate was only 44.3%. What’s more, 73.7% of the patients experienced at least 1 severe late toxicity. Consistent with this, Han et al. [9] reported an incidence of 70.3% of Grade 3–5 late toxicities after IMRT for recurrent NPC. About 69% of the patients’ deaths were attributed to radiation injuries. In our study, 54 (29.3%) patients died of radiation-related complications. Among them, 44 patients died of massive hemorrhage, represents the main cause of death. Seventy-five percent (33 out of 44) of these patients were locally advanced disease. All of the above results inform us that radiation induced injuries are still common even in the era of IMRT. We should pay more attention to make balance between the benefits of high-dose IMRT in disease control and the risk of severe late toxicities.

The pathogenesis of radiation-induced normal tissue injury is complex and involves different mechanism including DNA damage repair, inflammation, cell death, angiogenesis, matrix remodeling and so on. Stem cells (SCs) that are defined as the subset of cells with capability to self-renew and to produce more differentiated cells have shown significant implications in radiation-induced late toxicities in recent years [30]. Studies have shown that SCs can be a major target for genetic and epigenetic alteration leading to radiation-induced toxicity [31, 32]. The ability of resident SCs to reconstitute functional cells determines the onset and severity of the radiation injury [33, 34]. The use of stem cell therapy to promote recovery of normal tissues exposed to radiation is a new but burgeoning area of research [35]. The potential benefits of stem cell therapy include cell replacement, trophic support to the surrounding host tissue, protecting and restoring endogenous cell function and thus reducing normal tissue injury and hasten the recovery of patients [36–39]. Preclinical and early-stage clinical studies have shown encouraging therapeutic potential of stem cells for treating radiation-induced toxicities in different organs [35, 40–42]. However, successful translation to the clinic still faces many barriers including teratoma formation, immunorejection, disease progression, genomic stability and other ethical issues [35].

Mucosal necrosis and massive hemorrhage of the nasopharynx are the most severe late complications and also the leading cause of death after re-irradiation for recurrent NPC. The mechanism of mucosal necrosis is not well clarified. Marx [43] suggested the possible sequence: radiation causes formation of “3H”(hypoxic-hypovascular-hypocellular) tissue, in which the ability to replace normal collagen or cellular loss is severely compromised or non existent. This may eventually result in tissue disintegration and chronic unhealed wound. Rupture of radiation-induced internal carotid artery pseudoaneurysm is another cause of massive hemorrhage. The exact mechanism of pseudoneurysm formation is multifactorial. Radiation caused obstruction of vascular, premature atherosclerosis, adventitial fibrosis, and necrosis of the arterial wall. Combined with high blood pressure of the great vessel, it could result in the rupture of the arterial wall and even dissection with extravasation blood [44–47]. Other aggravating factors such as infection, previous surgery, trauma, underlying cardiovascular disease or hypertension can also accelerate the production or the rupture of pseudoaneurysm [46, 48]. Treatment of mucosal necrosis is difficult and there is no effective therapy for it. Hua et al. [49] tried to treat 28 nasopharyngeal necrosis patients with endoscopy surgery. The results showed that clinical symptoms (foul odor and headache) were relieved to various degrees in all patients and 8 patients were cured after surgery. However,9 (32%) patients died of sudden nasopharyngeal massive bleeding. Potential negative factors relate to necrosis include old age, co-morbidities such as diabetes, poor general condition and advanced tumor stage [9]. Tian et al. [50] recently found that disease-free interval between primary and re-irradiation (DFI) and recurrent GTV are also independent predictors for mucosal necrosis. Patients with a DFI ≤2 years or GTV > 30 cm^3^ has higher incidence of mucosal necrosis than those with a DFI > 2 years or GTV ≤ 30 cm^3^. More studies focusing on identifying high-risk populations of SAEs and optimal individualized treatment strategies are urgently needed.

The efficacy of chemotherapy for recurrent NPC, either as the sole treatment or combined with RT is still unclear [51]. Results from retrospective studies are less than satisfactory [51–53]. Collectively, the addition of chemotherapy may improve tumor response rate and local control, but no benefit in survival rates. As for locally advanced recurrent NPC, induction chemotherapy can be used to shrink the tumor to permit better target contouring for RT and to better protection for critical normal tissues [54]. In the study by Chua et al. [55] evaluating the effects of induction chemotherapy with cisplatin and gemcitabine before IMRT for locally recurrent NPC, 75% of the patients had partial response after induction chemotherapy and complete response was achieved in 61% of the patients after IMRT. The 1-year LRFS and OS rates were 75 and 88%, respectively. Further studies are on the way. In the large series reported by Chang et al. [51], 44.1% of the patients (82 out of 186 recurrent NPC patients) received cisplatin based chemotherapy in addition to RT. However, both univariate and multivariate analysis showed that the addition of chemotherapy did not significantly improve survival (22.5% vs. 22.8%, *p* = 0.904). In our study, chemotherapy was independent predictors for DMFS, but not for LRFS and OS. However, it is worthwhile to note that there was selection bias in our series since chemotherapy was usually given to patients with advanced disease or poor response to RT. Prospective randomized clinical trails are needed to further clarity the role of chemotherapy for recurrent NPC.

## Conclusion

Our long-term results show that re-irradiation with IMRT is an effective choice of treatment for patients with recurrent NPC. Severe late complications are major causes of death, especially for locally advanced disease. Studies focusing on the optimum balance between disease control and quality of life are extremely needed.

## Abbreviations

AJCC/UICC: American Joint Committee on Cancer//Union for International Cancer Control
CI: Confidence interval
CT: Computed tomography
CTV: Clinical target volume
DFI: Disease-free interval
DMFS: Distant metastases–free survival
ECT: Emission-computed tomography
GTV: Gross tumor volume
IMRT: Intensity modulated radiation therapy
LC: Local control
LRFS: Local recurrence–free survival
MRI: Magnetic resonance imaging
NCI-CTCAE: National Cancer Institute Common Toxicity Criteria for Adverse Events
NPC: Nasopharyngeal carcinoma
OAR: Organs at risk
OS: Overall survival
PRV: Planning organ-at-risk volume
PTV: Planning target volume
QOL: Quality of life
RT: Radiotherapy
SAE: Severe adverse effects
SIB: Simultaneous integrated boost
SPSS: Statistical Package for Social Sciences
TPS: Treatment planning system
TTR: Time to recurrence
